# The Kendrick modelling platform: language abstractions and tools for epidemiology

**DOI:** 10.1186/s12859-019-2843-0

**Published:** 2019-06-11

**Authors:** Mai Anh BUI T., Nick Papoulias, Serge Stinckwich, Mikal Ziane, Benjamin Roche

**Affiliations:** 1 Software Engineering Department, School of Information and Communication Technology, Hanoi University of Science and Technology, Hanoi, Vietnam; 2 Université de La Rochelle, UMR 7266 LIENSs, CNRS, La Rochelle, France; 3 Sorbonne Université, IRD, Unité de Modélisation Mathématiques et Informatique des Systèmes Complexes, UMMISCO, F-93143, Bondy, France; 4 Université de Yaoundé I, IRD, UMMISCO, Yaoundé, Cameroon; 5 Université de Caen Normandie, Caen, France; 6 Université de Paris, Paris, France; 7 Sorbonne Université, CNRS, Laboratoire d’Informatique de Paris 6, LIP6, F-75005, Paris, France

**Keywords:** Domain-specific language, Modularity, Mathematical modelling, Epidemiological modelling, Compartmental models

## Abstract

**Background:**

Mathematical and computational models are widely used to study the transmission, pathogenicity, and propagation of infectious diseases. Unfortunately, complex mathematical models are difficult to define, reuse and reproduce because they are composed of several concerns that are intertwined. The problem is even worse for computational models because the epidemiological concerns are also intertwined with low-level implementation details that are not easily accessible to non-computing scientists. Our goal is to make compartmental epidemiological models easier to define, reuse and reproduce by facilitating implementation of different simulation approaches with only very little programming knowledge.

**Results:**

We achieve our goal through the definition of a domain-specific language (DSL), Kendrick, that relies on a very general mathematical definition of epidemiological concerns as stochastic automata that are combined using tensor-algebra operators. A very large class of epidemiological concerns, including multi-species, spatial concerns, control policies, sex or age structures, are supported and can be defined independently of each other and combined into models to be simulated by different methods. Implementing models does not require sophisticated programming skills any more. The various concerns involved within a model can be changed independently of the others as well as reused within other models. They are not plagued by low-level implementation details.

**Conclusions:**

**Kendrick** is one of the few DSLs for epidemiological modelling that does not burden its users with implementation details or required sophisticated programming skills. It is also currently the only language for epidemiology modelling that supports modularity through clear separation of concerns hence fostering reproducibility and reuse of models and simulations. Future work includes extending Kendrick to support non-compartmental models and improving its interoperability with existing complementary tools.

**Electronic supplementary material:**

The online version of this article (10.1186/s12859-019-2843-0) contains supplementary material, which is available to authorized users.

## Background

The complexity of new infections, relying on many inter-connected factors [1–3] such as biodiversity decline [4], antibiotics resistance [5] or intensification of worldwide trade [6], makes the anticipation of their propagation and their evolution a challenge. Epidemiological models could help address this challenge. Indeed, mathematical and computational models have become widely used for investigating the mechanisms of infectious disease propagation [7, 8], exploring their evolutionary dynamics [9, 10], or informing control strategies [11, 12]. They largely rely on the so-called SIR framework [7, 13] where the host population is divided into compartments corresponding to the epidemiological status of individuals: those Susceptible to the pathogen (state *S*) can become Infectious (state *I*), allowing them to transmit the pathogen and eventually become Recovered (state *R*), i.e. immunised against the pathogen. Such models aim to characterise the transition between these categories and the consequences on the dynamics of each category, especially the ‘Infectious’ one that contains diseased individuals. Obviously, other categories can be added *ad libitum* in order to take into account different concerns (factors) such as the aforementioned ones.

Epidemiological models are sometimes analytically tractable and can more generally be implemented and simulated in different ways. The first approach is often to express the life cycle as a system of ordinary differential equations to be deterministically simulated through numerical methods, such as Runge-Kutta algorithms [14]. While this approach is especially useful to understand the average dynamics without chance, shifting to a stochastic approach (e.g., through Gillespie algorithms [15]) is known to be more realistic compared to real epidemiological data [7], which allows to analyze the impact of random events on the simulated dynamics of infectious diseases (such as their seasonality [7]). Finally, an agent-based implementation is sometimes required to reach a level of details that would not be tractable with other approaches because of combinatorial explosion [16].

Unfortunately, complex mathematical models are difficult to define, reuse and reproduce because their various concerns are intertwined. Secondly, implementing them requires various degrees of programming skills that span from rudimentary for the deterministic implementations to very sophisticated for agent-based models. Thirdly, implementation choices can be very heterogeneous with non-negligible impacts on the disease dynamics. For instance, despite their simplicity, deterministic models can show very different dynamics according to the algorithm being used [7]. Finally, implementation details are intertwined with the epidemiological concerns making it even harder to define, reuse or reproduce models.

We address these issues through the definition of a domain-specific language (DSL), Kendrick, that relies on a very general mathematical definition of epidemiological concerns. Domain-Specific Languages (DSLs) separate modelling and specification concerns (conceptual model) from implementation aspects (computational model). Contrary to General-purpose Programming Languages (GPLs), DSLs are higher-level languages that provide abstractions and notations that are directly-related to the concepts of the studied domain [17–19].

A DSL itself, however, does not separate the domain concerns from each other. In epidemiology, this represents a significant challenge because the various concerns cannot be completely independent of each other. For instance, if a spatial concern is added to a model it is precisely because it is expected that the rate of infection is not uniformly distributed across space. Therefore, the key issue is to provide a way to express the various concerns as independently as possible despite their inevitable interactions.

We solve this problem thanks to a set of mathematical definitions that are general enough to capture a large variety of epidemiological concerns and that support a three-step approach in which models are:

1. defined abstractly and independently of each other (using the KendrickModel keyword),
2. combined in any possible order (using the Composition keyword),
3. instantiated and correlated to each other (using the Scenario keyword).

The interactions between models only occur in the third step so that model definitions can be defined and reused independently of each other.

To illustrate the practical usage of our modelling language, we present in this paper two case studies on measles and mosquito-borne diseases. Our approach is carefully validated through statistical comparisons between our simulation results and well-established platform simulations in order to guarantee the link with the mathematical epidemiology literature. While parts of the underlying mathematical models and of the syntax of Kendrick have been presented elsewhere [20, 21], this is the first time the software itself is presented, through a step by step coverage of its implementation and usage.

## Implementation

This section sheds light on the mathematical model on which Kendrick relies, on the Kendrick language itself as well as on its implementation (for installation and experimentation details see the Results & Discussion section).

### Overview of the underlying mathematical model

We have adopted a stochastic viewpoint in which models are Continuous-Time Markov Chains (CTMCs). Models that are defined deterministically from differential equations can be interpreted from a stochastic viewpoint provided some assumptions about the probability distribution which is often assumed to be Poisson. Conversely, the stochastic models that we consider can be abstracted back to the deterministic ones.

Studying the literature on CTMCs lead us to define models and their concerns as stochastic automata [22]. We have then looked for an operator to combine concerns as freely as possible. The concept of Stochastic Automata Network (SAN) [23] seemed general enough to be reused. The automata of a SAN are combined by a tensor sum operator when the underlying Markov processes are CTMCs as in our case.

There are two ways in which stochastic automata may interact [23]:

- The rate at which a transition occurs may be a function of the state of a set of automata. These transitions are called *functional transitions* and their rates *functional rates*.
- A transition in one automaton may force one to occur in one or several other automata. The latter are called triggered transitions.

In epidemiological models, it is crucial to support functional rates while we have not met examples that required triggered transitions. We have focused on functional rates because they allow the description of the heterogeneities that, as it was mentioned previously, motivate the use of different concerns in the first place. It is crucial to be able to express, for instance, that the force of infection depends on the region where individuals live. The point is that this information is deferred, in Kendrick models, to a special phase (introduced by the *Scenario* keyword) that is separated from the definition of concerns. These definitions can then be more easily reused.

With our plattform, any given model can be run as a deterministic or stochastic simulation. The default deterministic solver is RK4 [14]. Gillespie’s direct and tau-leap methods are both available for stochastic simulations. Stochastic individual-based simulations can also be run by triggering events at the level of individuals [16].

### The Kendrick DSL

The goal of the DSL is threefold: a) provide an easy-to-use concise and readable syntax, b) hide implementation details as much as possible, and c) provide a way to keep the concerns separated (i.e. avoid textual dependencies between them).

With these goals in mind, our DSL defines the following linguistic (syntactical and semantic) entities: Epidemiological *Models*, *Compositions*, *Scenarios*, *Simulations* and *Visualizations*, which are exemplified in Table 1. Each of these entities can be defined as a separate component (with its own file if necessary) and reused several times in multiple projects by simply referring to its name. Moreover, all of the aforementioned entities can be extended (i.e. specialized). It is possible to introduce new properties (see the SEIR model in case study I below), as well as overriding some of them.

**Table 1:** Kendrick DSL entities (Two species influenza SIR example)

	

The text color is due to Smalltalk syntax

First, *Models* are defined as independently as possible from each other. They are characterized by epidemic concerns (such as SIR or similar schema, spatial aspects, age or sex structure...). These models can then be combined (through the *Composition* entity). *Scenarios* describe the initial conditions of experiments and can themselves be combined in different ways to provide inputs to *Simulation* entities. These entities define algorithmic properties for experiments, such as solvers and timing constraints. Finally, the *Visualization* entities define the desired visual outputs (such as epidemiological figures and maps) of the experimental results.

Functional rates that define the heterogeneities of the various concerns, and thus bind some of them, are introduced in the *Scenario* entities only. This is crucial to be able to define concerns as independently as possible. Note that the Kendrick syntax was very carefully designed to make it very convenient to name compartments of any size while the typical Matlab implementations heavily rely on low-level matrix operations. This notation is quite convenient to initialize parameters and especially functional rates whose initial values may depend on several concerns. See for instance the initialization of the ***mu*** and ***rho*** parameters in the Mosquito-borne disease case study further in this document. In this case, ***mu*** and ***rho*** are functional rates because their value depends on the species. More complex functional rates involving several concerns can also be easily initialized by assigning values to compartments of various sizes.

### Implementation details

Kendrick is implemented on top of the Pharo [24] programming environment using the Moose meta-modelling platform [25]. The numerical analysis back-end is based on the PolyMath framework [26] while the visualisation sub-system relies on Roassal [27, 28] and the UI components on GT Tools and the Moldable Inspector [29]. In addition to that, the DSL extensively relies on Pharo’s reflective sub-system [30], with extensions based on the PetitParser [31] framework and on the Helvetia parsing workbench [32].

Kendrick supports all major desktop platforms (Gnu/Linux, MacOS and MS Windows) and adopts an open continuous integration process (based on github^1^ and Travis CI^2^). It has been extensively tested using the SUnit [33] framework, readily available through a dedicated website^3^ and well documented through a collaborative wiki^4^.

## Results

In this section, we first provide practical information on how to install and start using Kendrick. Then we illustrate in detail how to simulate the evolution of two models of infectious diseases. The first one is measles, a childhood disease that has been extensively studied through epidemiological modelling while the second model is a vector-borne disease, which allows us to illustrate the use of a multi-host concern.

### Installing & running Kendrick

The easiest way to install Kendrick is to download a pre-compiled bundle for your platform from Github^5^. After unzipping the corresponding compressed archive file, simply run one of the Kendrick launchers (**KendrickUI** for Mac and Linux, or **KendrickWinLauncher** for Windows).

Alternatively, a very straight-forward method to compile Kendrick from sources is provided on all systems with a bash command-line (including Linux, Mac and Windows with Cygwin and/or the Windows 10 Bash sub-system), by issuing the following command:


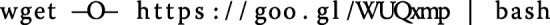


This command will automatically retrieve all the required dependencies, compile Kendrick from sources and set up all the execution scripts for normal or development use.

Kendrick can be used in three different modes. First, the UI mode relies on a specific IDE to edit and run Kendrick models. This UI follows the Cascading Lists^6^ pattern of navigation: each selection reveals a new column of actionable information (on the right), with a horizontal bar on the bottom that controls the position and size of the viewport. This mode is launched by invoking the following command.


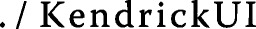


Second, Kendrick can be used from the command-line. Kendrick models are edited using any source code editor and stored in the Sources directory of the Kendrick installation, along with all the other models. A model is run using the following command and the results are then stored in the Output directory.


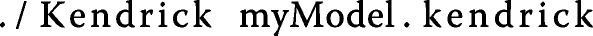


Third, an advanced UI mode is available where Kendrick is run with the full development environment (allowing to use both the DSL and the Pharo API of Kendrick). This mode is run this way:


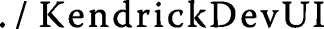


### Case study I: Measles

We consider the transmission of measles through a SEIR model with demography [34]. Individuals are born in the *Susceptible (S)* status with a birth rate *μ*, then may become *Exposed (E)* (i.e. infected but not yet infectious) with transmission rate *βI*. After an average latent period of 1/*σ*, they may become *Infectious (I)* and transmit the pathogen. Finally, they may move to the *Recovered (R)* class after an average infectious period of 1/*γ*.

**Fig. 1:**
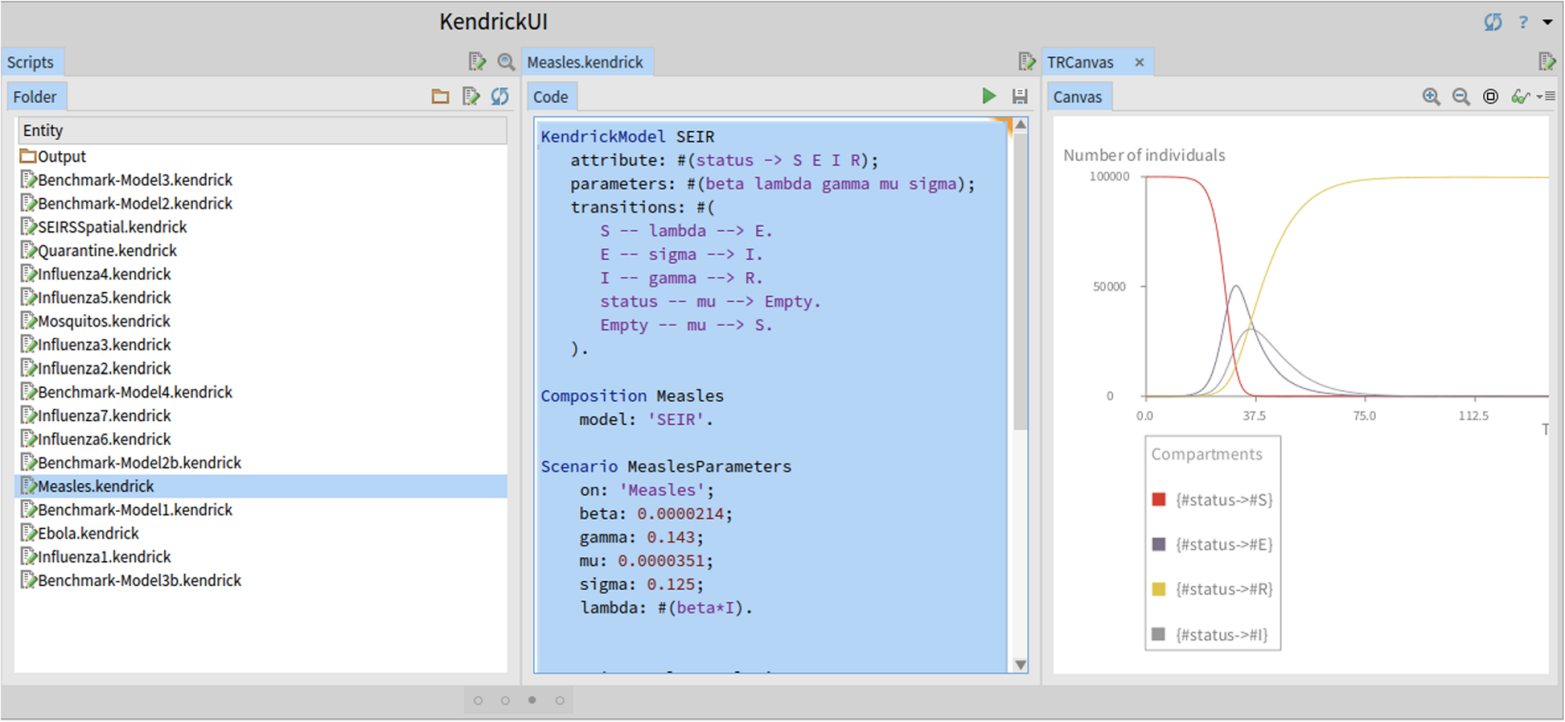
Kendrick UI: Running the Measles script


The model, shown below, is available from the Kendrick UI by navigating either to Scripts/Measles.kendrick (as a single file script, see Fig. 1) or to Projects/Measles.

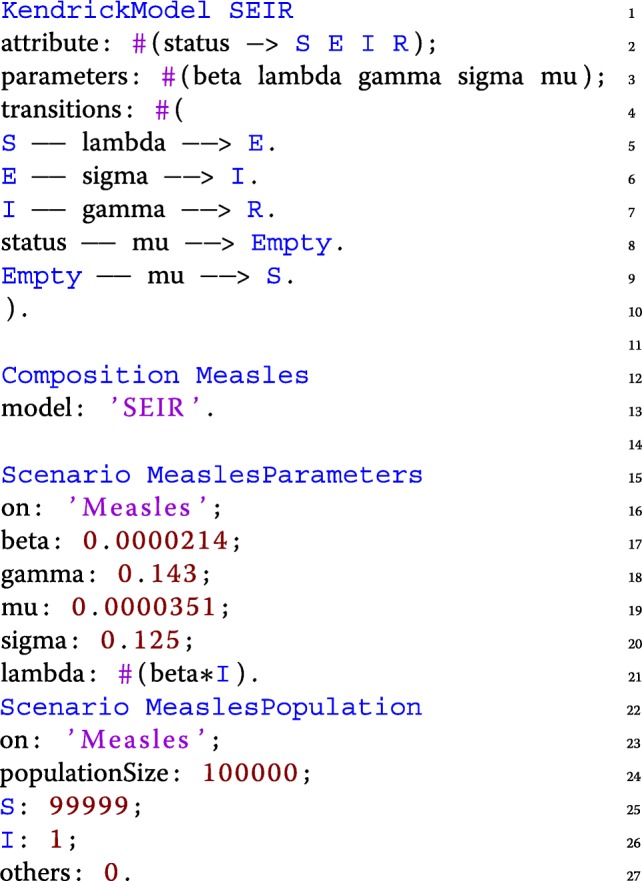


Lines 1 to 9 define the core epidemiological concern, SEIR, including the transition parameters. Complete models (such as Measles) are produced by a *Composition* which computes a tensor sum of one or several modelling concerns (lines 12-13). In this simple example, the Measles model includes a single concern: SEIR.

Finally, from lines 15 to 28, two *Scenarios* are declared separately to foster reuse even though both are required to run the Measles model. The first one assigns a value to each parameter of the model while the second one sets the initial value of each compartment of the core concerns (S, E, I, and R).

This version of the Measles model relies on the transition syntax of Kendrick, but ODEs (Ordinary Differential Equations) can also be used to express the same model:


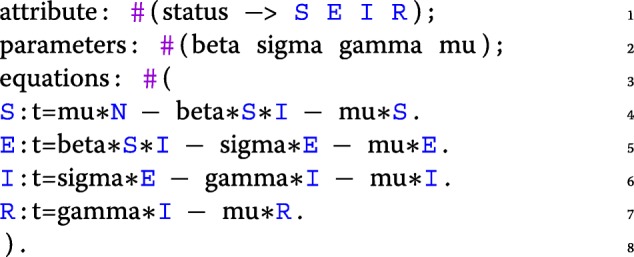


A core epidemiological concern (e.g., SIR, SEIR etc.) is required in all epidemiological models. Defining them as separated entities fosters their reuse in different models. The most frequently used entities are gathered in the standard Kendrick library (available through our editor by navigating to Library/KendrickModel.

**Fig. 2:**
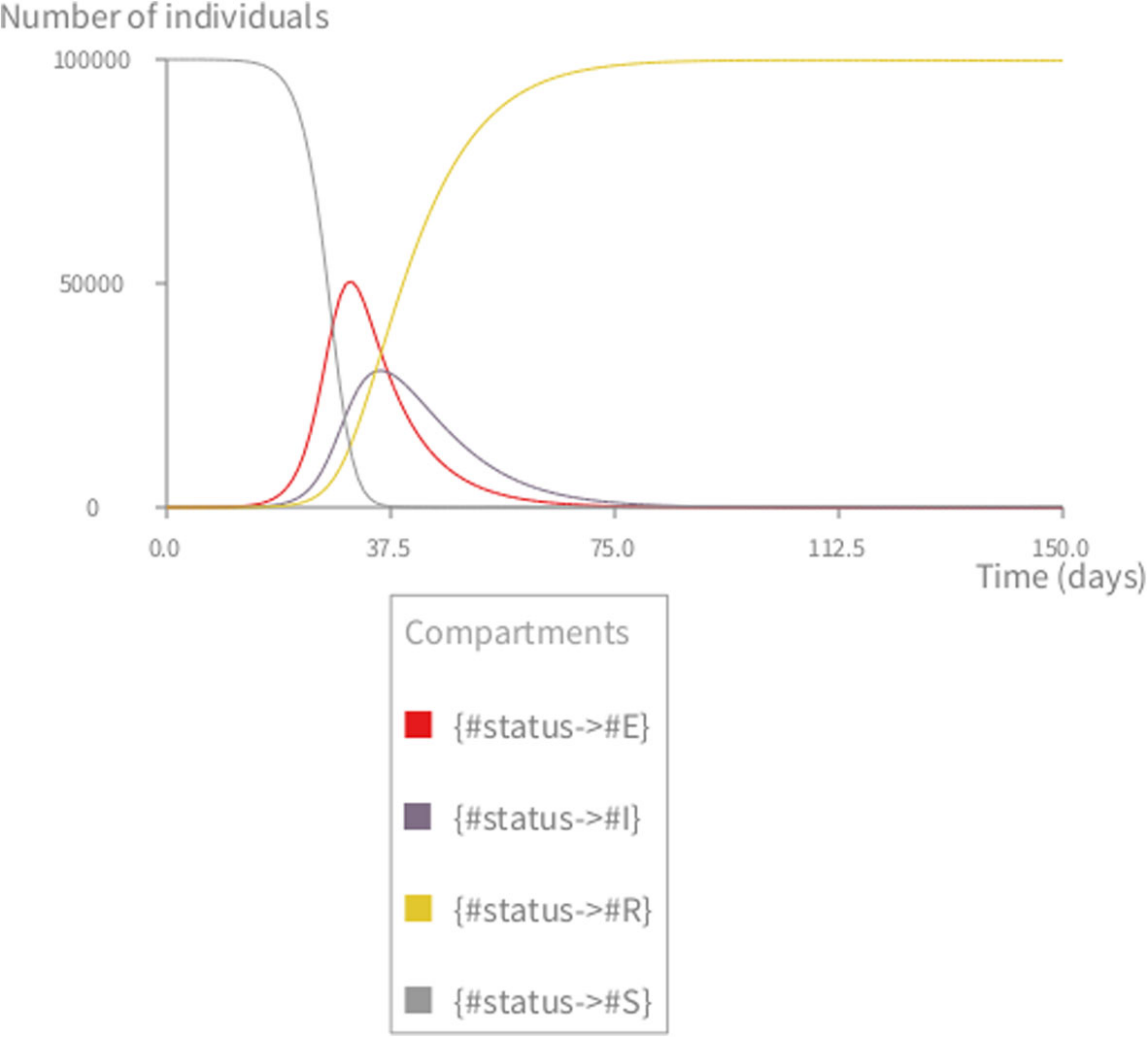
Deterministic simulation of the measles model. *S*=99999,*E*=0,*I*=1,*R*=0,*β*=0.0000214, 1/*γ*=7 days, 1/*σ*=8 days, *μ*=1/(78∗365) in *day*^−1^,*N*=100000. The graph shows the infectious deterministic dynamics of the measles model using the KENDRICK language


The initial size of the population is 100,000 individuals. The value of the other parameters are taken from the related literature [7, 34]. Figure 2 shows the result of the deterministic simulation (using the RK4 solver) produced by the *Simulation* and *Visualization* entities shown below:

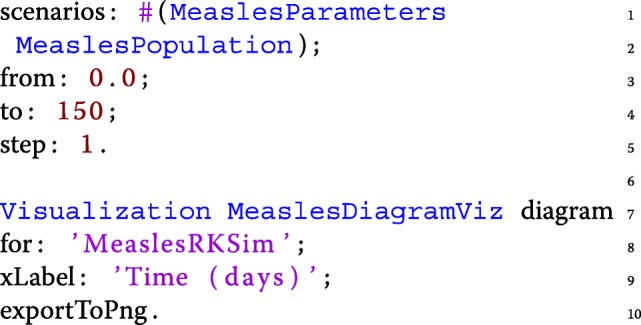


The *Simulation* entity (from lines 1 to 5) uses both the scenarios that were previously defined, and specifies which algorithm to use (here Runge-Kutta) and the timing characteristics (time step and duration of the simulation). Finally, the *Visualization* entity specifies the desired output for a specific *Simulation* and by default plots the dynamics of the infected compartment.

### Case study II: A mosquito-borne disease with three host species

Our second example is an SIR model with demography of a mosquito-borne disease with three host species. These species are a vector (*mosquito*) and two potential hosts: *reservoir1* and *reservoir2*. So the population that is always partitioned using at least the *status* attribute is here also partitioned using the *species* one leading to 3x3 compartments.

All the species have the same six transitions: birth, deaths (for each one of the three status compartments), infection and recovery. Given a transition, the transition function is the same for all states. Only 4 generic transitions need to be defined with Kendrick, which will then automatically generate the remaining ones for each species of the model.

**Fig. 3:**
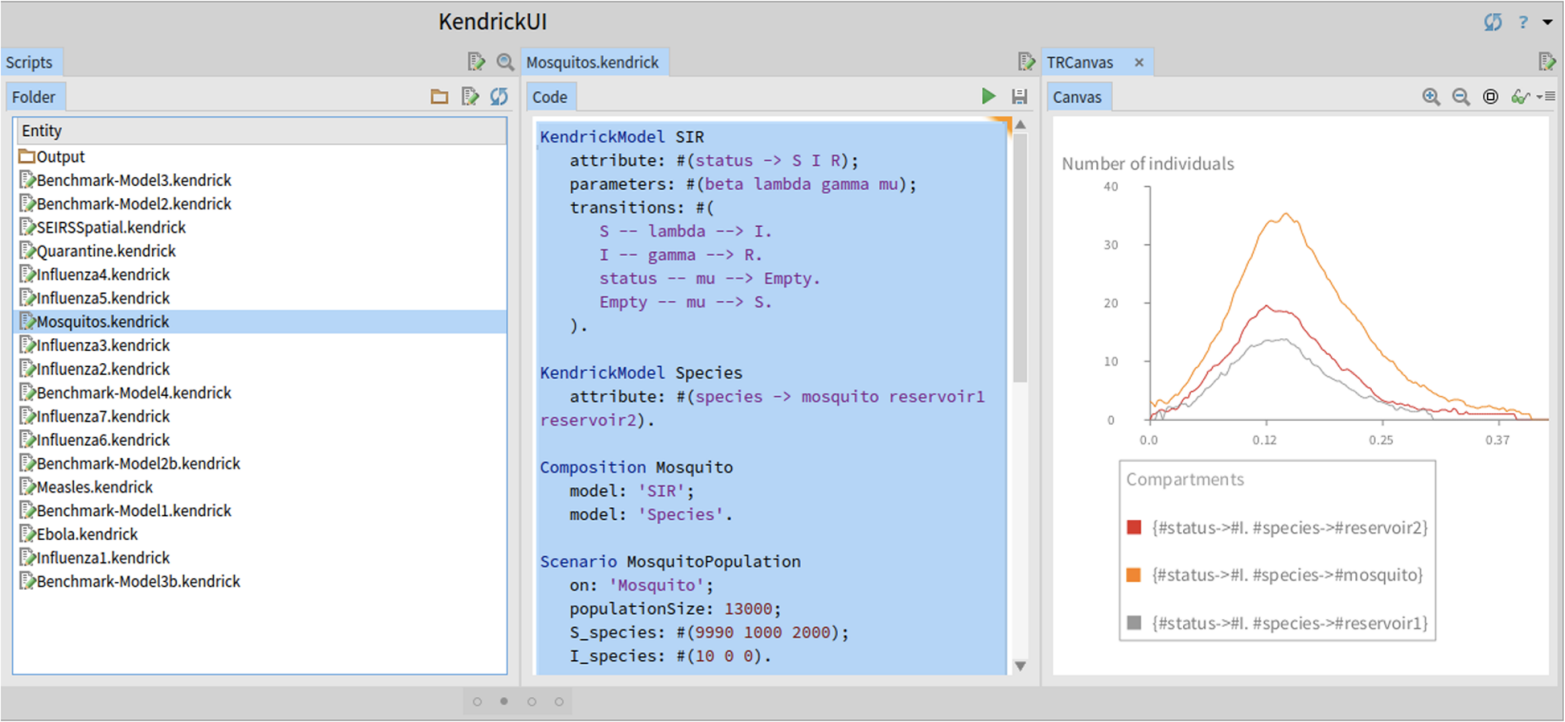
Kendrick UI: Running the mosquitos script

As previously, the model is available through the Kendrick distribution by navigating through our editor either to Scripts/Mosquitos.kendrick (single file script, seen in Fig. 3) or to Projects/Mosquito.

The script of the model is shown below.


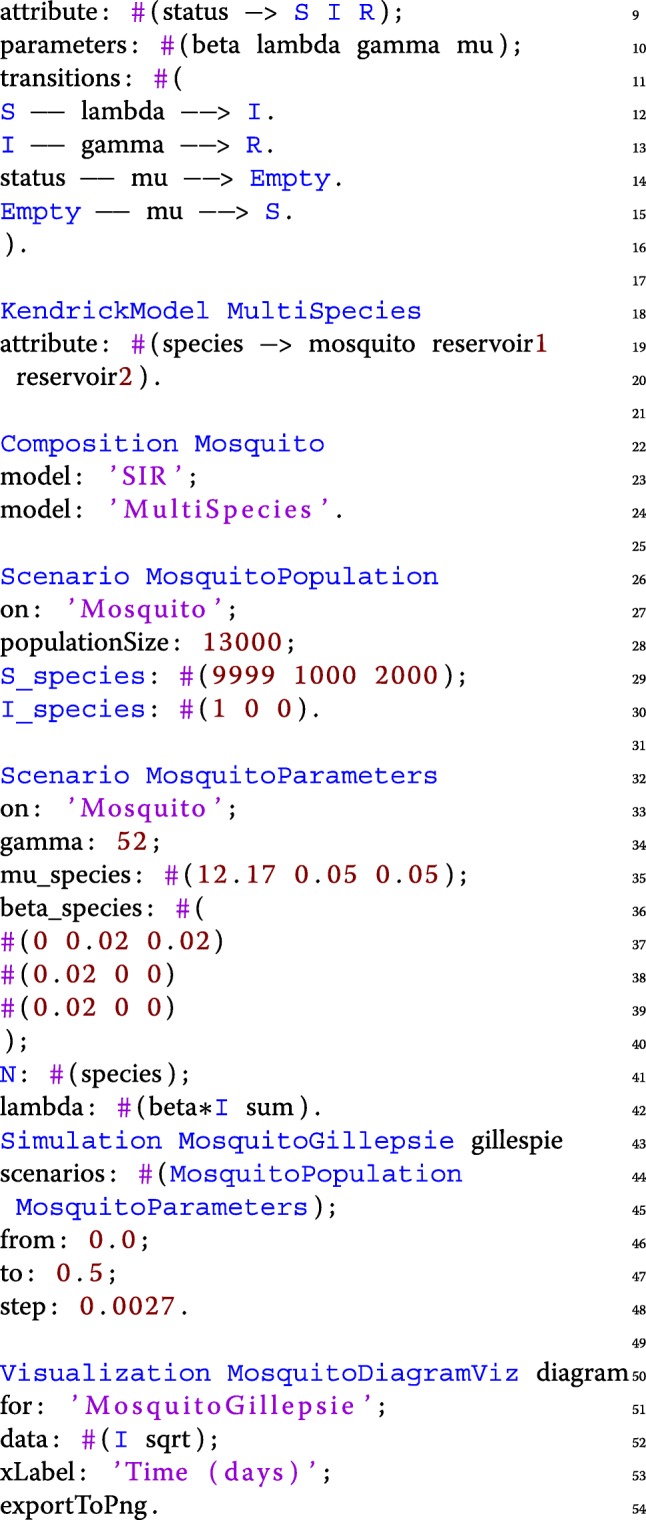


The SIR concern is defined (from lines 1 to 9) including transitions and parameters. The multi-species concern (lines 11-12) includes the three aforementioned species and has no transitions since individuals do not change their species. The Mosquito model combines the epidemiological and multi-species concerns (lines 14 to 16).

The Mosquito model is then complemented by two scenarios. The (*MosquitoPopulation*) scenario (lines 18 to 22) sets the initial value of the compartments while the second scenario (*MosquitoParameters*) sets the value of the parameters (lines 24 to 34). When the initial size of a compartment depends on the species a compound name is used such as "mu_species" or "beta_species" and a vector is then provided with a value for each species rather than a single value for all the species. *R* is not mentioned because its value is the default one (zero).

As mentioned earlier, when the value of a parameter not only depends on the state of its own automaton (i.e. concern) but on the state of another one it is expressed as a *functional rate* which is the case for **mu** and **beta** (lines 27-32). Both parameters are defined by the SIR concern but depend on the *species*. Compound names are used in this case too to specify vectors or even matrices. Mu is initialized by a mere vector because In order to specify *functional rates*, parameters are followed by attribute keys to denote how their values vary with such attributes, i.e. line 27 means that the value of parameter **mu** varies with *species*, the first value represents **mu** of *mosquito*, the second one is of *reservoir1* and the last one is of *reservoir2*.

**Fig. 4:**
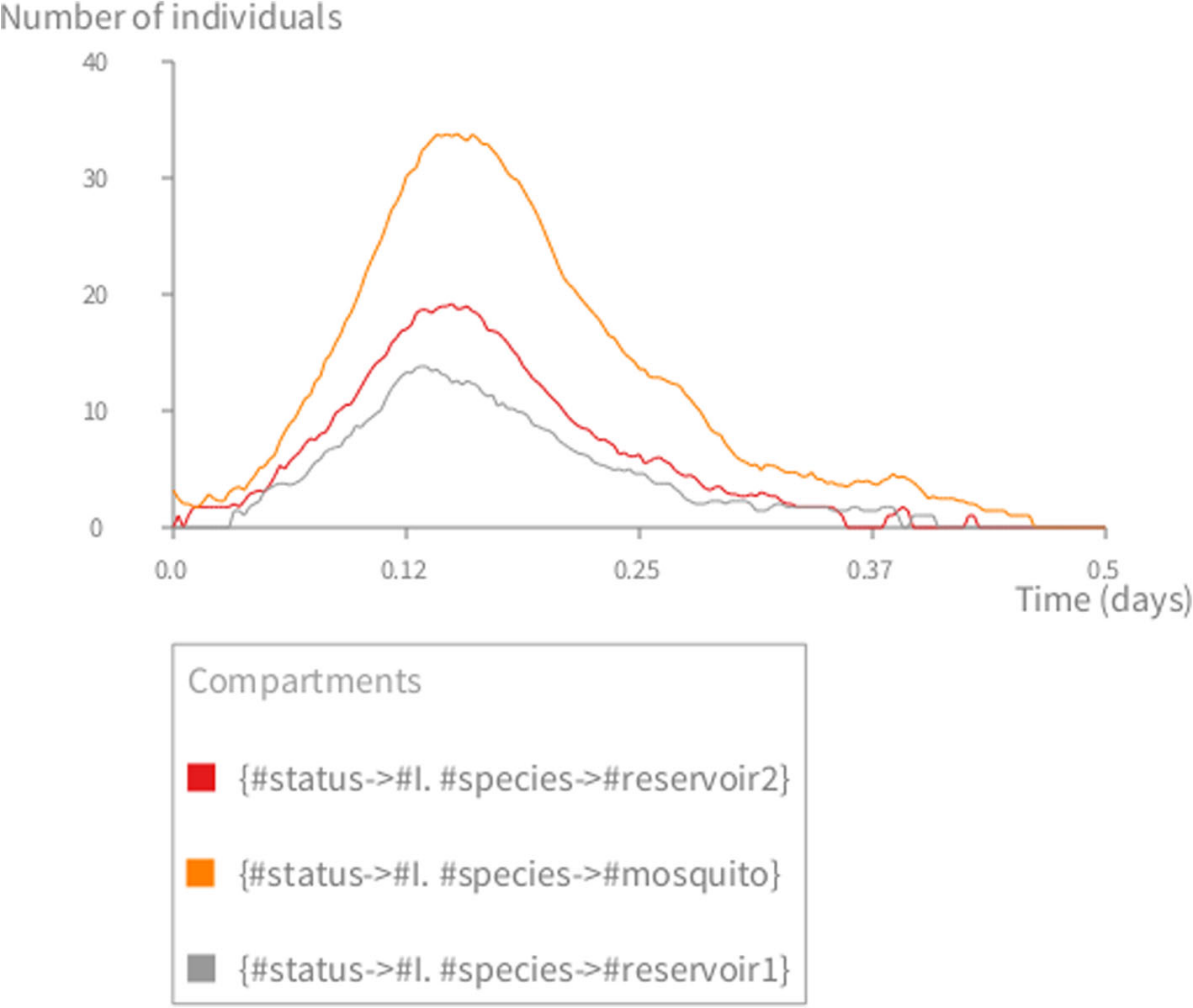
Stochastic simulation of the mosquito-borne disease. The multi-host model with three species: mosquito, reservoir1, reservoir2. *S*_1_=9999 (mosquito), *S*_2_=1000 (reservoir 1), *S*_3_=2000 (reservoir 2); *I*_1_=1,*I*_2,3_=0,*R*_1,2,3_=0,*N*_1_=10000,*N*_2_=1000,*N*_3_=2000; *σ*=52,*μ*_1_=365/30,*μ*_2,3_=1/20,*β*_12_=*β*_13_=0.02,*β*_*others*_=0.0

The simulation (lines 36 to 40) runs the Gillespie algorithm for the given time-frame and step using both the aforementioned scenarios. The visualization (lines 42 to 46) plots the dynamics of the infectious (I) compartment (Fig. 4).

### More example models are available

The first case-study focused on a basic model with a single (core) concern: SEIR. The second case-study included a core concern (SIR) and a multi-species one. More example models combining more concerns (such as spatial, control policies, sex or age structures, multi-strain, etc.) are available in the Kendrick distribution and on the wiki^7^.

For example, the Influenza6 model (see *Scripts/Influenza6.kendrick* in the distribution or^8^ on the wiki) includes SEIRS, a multi-species concern, a quarantine concern, and a spatial concern of 5 countries of Southeast Asia. More complex spatial graphs can be constructed by importing data from external sources (e.g., GIS data, flight connections etc.).

The diversity of these examples suggest that Kendrick can support the most common epidemiological concerns.

### Batch analysis & integration with external tools

In order to support batch exploration of epidemiological models (for sensitivity analysis for example), *epidemiological experiments* can be defined with different parameter values to explore a grid of the parameter space.

**Fig. 5:**
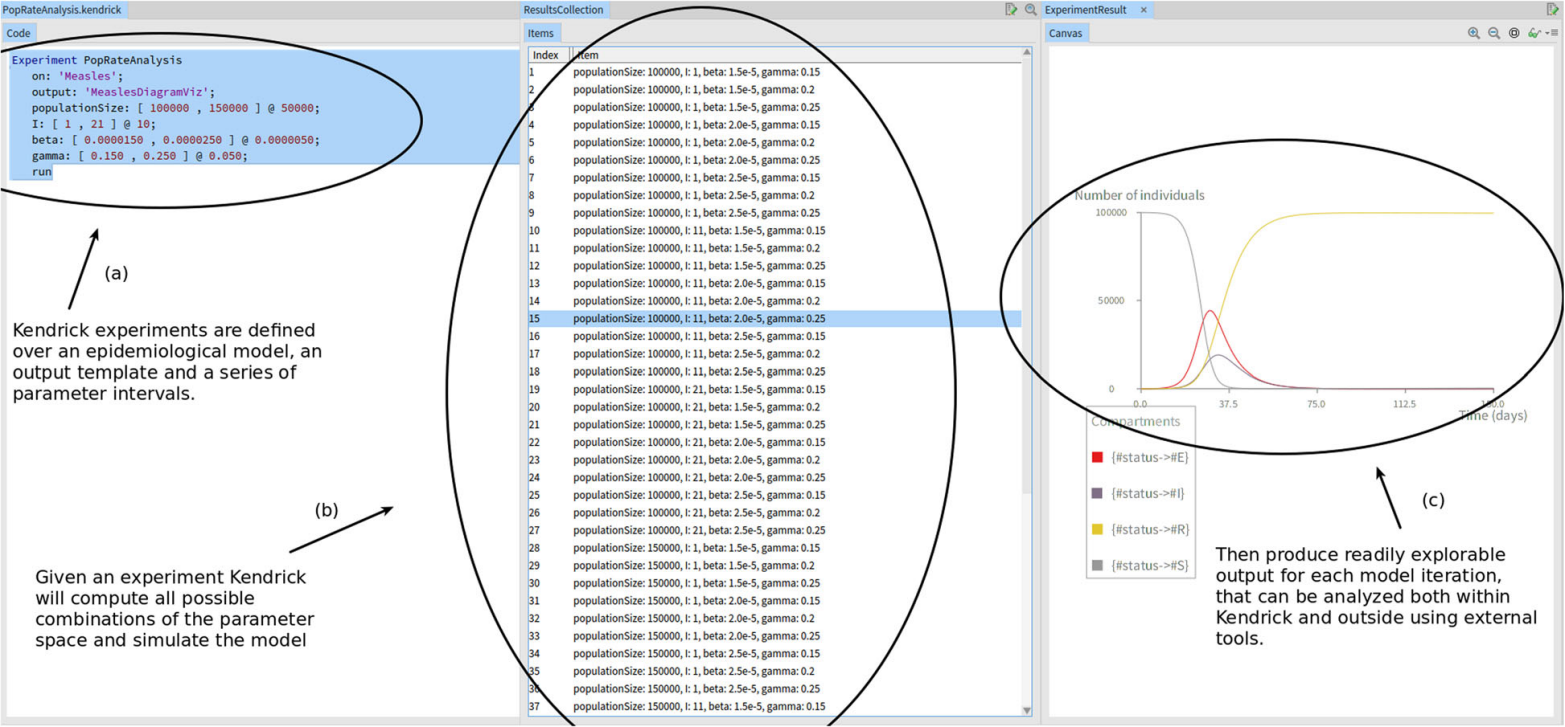
Declaring an epidemiological experiment for the Measles model and exploring the results within the Kendrick platform

These experiments can be defined using either **(a)** the dedicated *Experiment* entity in Kendrick Projects (as shown in the code-snippet below and in Fig. 5) or **(b)** command-line arguments.

In both cases, the results of the experiments can be analysed by third-party software. In the case of the command-line experiments, the set-up allows Kendrick to be driven by and integrated with platforms such as R^9^ or OpenMole^10^ for further analysis.


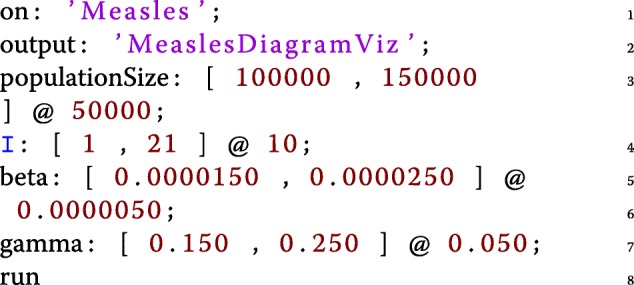


An experiment, *PopRateAnalysis* is defined on the *Measles* model using the *MeaslesDiagramViz* visualization as an output template (lines 1-3). A series of parameter intervals of the form *[min, max] @ step* are then introduced (lines 4-7). *populationSize* will thus get 2 values: 100000 and 150000, while the initial infected population as well as *γ* and *β* will get 3 values each. Finally, line 8 runs the experiment.

Kendrick runs a simulation for each of the 54 (2x3x3x3) combinations. The results can then be explored and analysed as seen Fig. 5. The results are also exported automatically in the Output folder of the project for further analysis with external tools.

Model fitting to observed data is possible by running a Kendrick model from the command line. An example is given on our development website^11^ where a complete Kendrick model is built in Matlab/Octave^12^. The space of the possible parameter values is then explored, driven by a minimization function written in Octave. More direct support by Kendrick is planned to minimize the need of external tools.

### Validating the implementation

To validate the core functionalities of Kendrick as a modelling and simulation platform, the output of deterministic Kendrick models have been compared to a reference implementation of the same models on Scilab [35]. The simulation results (see Fig. 2) suggest that for deterministic models, Kendrick results are identical to those of the reference implementation.

**Fig. 6:**
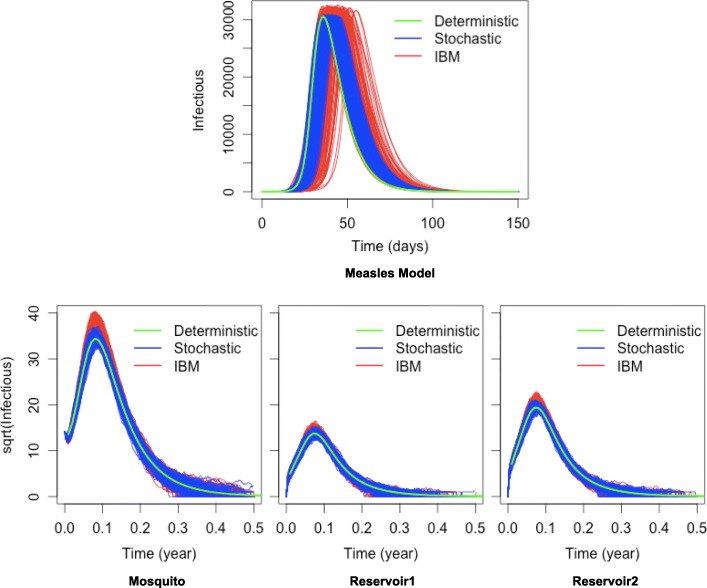
Comparison between the dynamics of deterministic, stochastic and individual-based model. We show the simulation results of two models in three formalisms: deterministic (green line), stochastic (blue lines) and individuals-based (red lines). The first row shows the results of the measles model. The second row shows the results of the mosquito-borne model with three host species

The dynamics of deterministic simulations have also been compared to those of Gillespie and individual-based simulations (Fig. 6).

The results for the measles model can be seen in the upper part of Fig. 6, with the lower part displaying the results of the mosquito-borne model for each individual host species. The deterministic dynamics can be superimposed on the stochastic and individual-based ones, validating our implementation of the simulation logic.

Finally, outputs of individual-based and stochastic simulations using the same configuration have been cross-examined. Key properties of the epidemiological dynamics, such as the duration and the peak of the epidemic, have been extracted from the results of 200 executions of each individual-based and stochastic model. A Kolmogorov-Smirnov test on each pair of samples has showed no statistical difference between the individual-based simulations and the stochastic ones. The results can be seen in Table 2, where all *P*−*values* are greater than 0.05, validating that the resulting distributions are statistically indistinguishable. This suggests that our individual-based and stochastic algorithms are correctly implemented and provide compatible results.

**Table 2:** *P*-values of Kolmogorov-Smirnov test on two models over some disease global properties

Model		Global properties of diseases	
		Peak of epidemic	Epidemic duration
Measles		0.1777	0.3275
Mosquito-borne disease	Species 1	0.7920	0.7112
	Species 2	0.6272	0.8643
	Species 3	0.7920	0.2202

## Discussion

Although DSLs have been used before in the context of bioinformatics [36–38], only a small number of them focused on epidemiological modelling [39, 40]. For example, *Ronald* [39] is a DSL for studying the interactions between malaria infections and drug treatments, but has unfortunately been discontinued. Schneider and al. [40] have also proposed a DSL for epidemics, but their solution only support agent-based models. Mathematical modelling languages (MMLs) such as Scilab [35], Modelica [41], Matlab [42] or JSim [43] do allow easier definition of mathematical models as sets of ODEs, but are too broad in scope to properly cover the domain-specific needs of epidemiology. On the other hand, individual libraries targeting epidemiological modelling, such as Epipy - a visualisation data tool for epidemiology written in Python [44], or GillespieSSA - an R package for generating stochastic simulation using Gillespie’s algorithms [45], cover only specific epidemiological needs.

Closer to our approach are computational modelling tools for epidemiology such as FluTe [46], GLEAMviz [47], STEM [48] and FRED [49]. These solutions use dedicated approaches to model the transmission of infectious disease and provide a graphical user interface (GUI) to specify and visualise an epidemiological model. The main features of these tools are summarised in Table 3 and compared to Kendrick.

**Table 3:** Modelling and simulation tools for epidemiology

Criteria		Kendrick	FluTe	GLEAMviz	STEM	FRED
Modelling approach	Deterministic	+	-	-	+	-
	Stochastic	+	-	+	+	-
	Individuals-based	+	+	-	-	+
Population	Multi-species	+	-	-	+	-
	Heterogeneous	+	+	-	-	+
Spatial Structure	Patch model	+	+	+	+	-
	Network of contact	+	-	-	-	+
	Mobility pattern	+	+	+	+	+
Intervention Strategies		+	-	+	+	+
Simulation at global scale		-	-	+	+	+

Contrary to solutions that only focus on stochastic simulations (such as GLEAMviz [47] and STEM [48]) our platform can provide more modelling options thanks to the availability of individual-based simulation. The large-scale epidemic simulations that can be carried out with GLEAMviz [47], STEM [48] or FRED [49] can also be considered with Kendrick since it provides all the necessary building blocks (disease spread, air-flight graph linking the airports) but it has not been attempted yet. A case study of meta-population model can be found in the Additional file 1 provided with this publication. This particular case is also an example of spatial visualisation integrated within the platform.

Given the above comparison, Kendrick can be considered as a higher-level disciplined solution that aims to cover as many specificities of epidemiological modelling as possible. The fact that Kendrick chooses the right level of abstraction for each case is also expected to help modellers focus on what is essential and avoid irrelevant or inconsistent definitions. This allows easier cross-examination of different modelling simulation schemes (such as deterministic, demographic-stochastic and individual-based). In particular, letting modellers directly manipulate epidemiological concepts, such as compartments, is expected to reduce the burden of having to deal with implementation details that typically involve programming-language-dependent matrix manipulations.

Currently, our platform has nevertheless several limitations. At the moment, we only support deterministic, stochastic and individual-based simulations. More high-resolution models like agent-based simulation where agents can have complex behaviours or contact-networks will be defined in the future. Spatial-based models based on partial differential equations (PDE) are also beyond the scope of this paper.

Furthermore, there is also an inherent trade-off in our pervasive use of a DSL, especially regarding simulations. While we do allow for the configuration of basic simulation parameters, there is currently no way to adapt simulations in more elaborate ways, without getting back to development in the host language (Pharo in our case). This is an acceptable limitation for a DSL in general, which aims to be user-friendly and re-usable, forfeiting the ability to control every little detail of the implementation by its end-users.

## Conclusion

This paper has introduced Kendrick, a language and a tool to make it easier to define, reuse and reproduce compartmental epidemiological models. Kendrick relies on a very general mathematical definition of epidemiological concerns as stochastic automata that are combined using tensor-algebra operators. A large class of epidemiological concerns can be defined this way, including multi-species, spatial concerns, control policies and sex or age structures. Concerns can be defined independently of each other, and combined into models that are simulated by different methods with very little programming knowledge.

Kendrick features have been highlighted using two classic examples of epidemiological models. The results produced using Kendrick are equivalent to well-established but harder-to-use, programming platforms. Kendrick supports batch-analysis and experimentation, allowing its combination with external tools for statistical analysis.

We hope that Kendrick will be further adopted and extended to support even more facets of epidemiology. It is available as open source software under the MIT Licence: http://ummisco.github.io/kendrick/.

## Availability and requirements

**Project name:** KENDRICK

**Project home page:** http://ummisco.github.io/kendrick/

**Operating System:** multi-platform (Linux/MacOS/Windows)

**Programming environment:** Pharo 6.1: http://www.pharo.org/

**Programming language:** Smalltalk

**Requirements:** All the required tools for the installation of KENDRICK are described on the project home page

**License:** MIT License

**Any restrictions to use by non-academics:** no restrictions

## Additional file

Additional file 1 A spatial SIR model specified with Kendrick. (PDF 348 kb)

## Abbreviations

DSLs: Domain specific languages
GPLs: General-purpose programming languages
GUI: Graphical user interface
MMLs: Mathematical modelling languages
ODEs: Ordinary differential equations
UI: User interface

## Footnotes

1
https://github.com/UMMISCO/kendrick

2
https://travis-ci.org/UMMISCO/kendrick

3
http://ummisco.github.io/kendrick/

4
https://github.com/UMMISCO/kendrick/wiki

5
https://github.com/UMMISCO/kendrick

6
http://designinginterfaces.com/firstedition/index.php?page=Cascading_Lists

7
https://github.com/UMMISCO/kendrick/wiki

8
https://github.com/UMMISCO/kendrick/wiki/Example-4.5:-Model-5---SEIRS-Multi-species-Multi-strains-Spatial-model

9
https://www.r-project.org/

10
https://openmole.org/

11
https://github.com/UMMISCO/kendrick/wiki/Fit-a-Kendrick-model-to-data

12
https://www.gnu.org/software/octave/

## References

[1] Cohen ML (2000). Changing patterns of infectious disease. Nature.

[2] Cevallos W, Ponce K, Levy K, Bates SJ, Scott JC, Hubbard A, Vieira N, Endara P, Espinel M, Trueba G, Eisenberg JNS (2006). Environmental change and infectious disease: how new roads affect the transmission of diarrheal pathogens in rural Ecuador. Proc Natl Acad Sci.

[3] Ostfeld RS, Keesing F (2012). Effects of host diversity on infectious disease. Ann Rev Ecol Evol Syst.

[4] Keesing F (2010). Impacts of biodiversity on the emergence and transmission of infectious diseases. Nature.

[5] Jones KE, Patel NG, Levy MA, Storeygard A, Balk D, Gittleman JL, Daszak P (2008). Global trends in emerging infectious diseases. Nature.

[6] Laxminarayan R (2013). Antibiotic resistance - the need for global solutions. Lancet Infect Dis.

[7] Keeling MJ, Rohani P (2008). Modeling infectious diseases.

[8] Xia Y, Bjornstad ON, Grenfell BT (2004). Measles metapopulation dynamics: a gravity model for epidemiological coupling and dynamics. Am Nat.

[9] Gandon S, Mackinnon MJ, Nee S, Read aF (2001). Imperfect vaccines and the evolution of pathogen virulence. Nature.

[10] Read AF, Huijben S (2009). Perspective: Evolutionary biology and the avoidance of antimicrobial resistance. Evol Appl.

[11] Bauch CT, Szusz E, Garrison LP (2009). Scheduling of measles vaccination in low-income countries: Projections of a dynamic model. Vaccine.

[12] Levin A, Burgess C, Garrison LP, Bauch C, Babigumira J, Simons E, Dabbagh A (2011). Global eradication of measles: an epidemiologic and economic evaluation. J Infect Dis.

[13] Anderson RM, May RM (1991). Infectious Diseases of Humans: Dynamics and Control.

[14] Griffiths DF, Higham DJ (2010). Numerical Methods for Ordinary Differential Equations.

[15] Gillespie DT (1977). Exact stochastic simulation of coupled chemical reactions. J Phys Chem.

[16] Roche B, Drake JM, Rohani P (2011). An agent-based model to study the epidemiological and evolutionary dynamics of influenza viruses. BMC Bioinformatics.

[17] Van Deursen A, Klint P, Viser J (2000). Domain-specific languages: An annotated bibliography. ACM SIGPLAN Not.

[18] Mernik M, Heering J, Sloane AM (2005). When and how to develop domain-specific languages. ACM Comput Surv.

[19] Fowler M (2010). Domain-specific Languages.

[20] BUI TMA, Stinckwich S, Ziane M, Roche B, HO TV. KENDRICK: A Domain Specific Language and platform for mathematical epidemiological modelling. In: the 11th IEEE RIVF International Conference on Computing & Communication Technologies-Research, Innovation, and Vision for Future (RIVF). IEEE: 2015. p. 132–7.

[21] Bui TMA, Papoulias N, Ziane M, Stinckwich S (2016). Explicit composition constructs in dsls: The case of the epidemiological language kendrick. Proceedings of the 11th Edition of the International Workshop on Smalltalk Technologies, IWST’16.

[22] Bui TMA, Ziane M, Stinckwich S, Ho TV, Roche B, Papoulias N (2016). Separation of concerns in epidemiological modelling. Companion Proceedings of the 15th International Conference on Modularity, MODULARITY Companion 2016.

[23] Plateau B, Stewart WJ (2000). Stochastic automata networks. Computational Probability.

[24] Black AP, Ducasse S, Nierstrasz O, Pollet D, Cassou D, Denker M. Pharo by Example. Kehrsatz: Square Bracket Associates; 2009, p. 333. http://pharobyexample.org/.

[25] Girba T. The Moose Book. Switzerland: Self Published; 2010. http://www.themoosebook.org/book.

[26] PolyMath. Open Source Software for Numerical Computation in Pharo. https://github.com/PolyMathOrg/PolyMath.

[27] Araya VP, Bergel A, Cassou D, Ducasse S, Laval J. Agile visualization with Roassal. Deep Into Pharo. 2013;:209–39.

[28] Bergel A (2016). Agile visualization.

[29] Chris A. Moldable tools. PhD thesis. University of Bern. 2016.

[30] Foote B, Johnson RE. Reflective facilities in Smalltalk-80. In: ACM Sigplan Notices. Vol. 24, No. 10. ACM: 1989. p. 327–35.

[31] Renggli L, Ducasse S, Gîrba T, Nierstrasz O (2010). Practical dynamic grammars for dynamic languages. The 4th Workshop on Dynamic Languages and Applications (DYLA 2010).

[32] Renggli L, Gîrba T, Nierstrasz O (2010). Embedding languages without breaking tools. European Conference on Object-Oriented Programming.

[33] Ducasse S. SUnit Explained. http://www.iam.unibe.ch/ducasse/Programmez/OnTheWeb/SUnitEnglish2.pdf.

[34] Anderson RM, May RM (1991). Infectious Diseases of Humans, vol. 1.

[35] Scilab. Open Source Software for Numerical Computation. http://www.scilab.org/.

[36] Fall A, Fall J (2001). A domain-specific language for models of landscape dynamics. Ecol Model.

[37] Degenne P, Lo Seen D, Parigot D, Forax R, Tran A, Ait Lahcen A, Curé O, Jeansoulin R (2009). Design of a domain specific language for modelling processes in landscapes. Ecol Model.

[38] van Engelen RA (2001). Atmol: A domain-specific language for atmospheric modelling. CIT J Comput Inf Technol.

[39] Antao T, Hastings IM, McBurney P. Ronald: A Domain-Specific Language to Study the Interactions Between Malaria Infections and Drug Treatments. In: Proceedings of the International Conference on Bioinformatics & Computational Biology (BIOCOMP 2008), Vol 2. CSREA Press: 2008. p. 747–52.

[40] Schneider O, Dutchyn C, Osgood N. Towards frabjous: a two-level system for functional reactive agent-based epidemic simulation. In: Proceedings of the 2nd ACM SIGHIT International Health Informatics Symposium. ACM: 2012. p. 785–90.

[41] Modelica Language. https://www.modelica.org/.

[42] Matlab. the Language of Technical Computing. http://www.mathworks.com/products/matlab/.

[43] Introduction of JSim Framework. http://www.physiome.org/jsim/.

[44] Epipy. Python tools for epidemiology. http://cmrivers.github.io/epipy/.

[45] GillespieSSA. Gillespie’s Stochastic Simulation Algorithm (SSA). http://cran.r-project.org/web/packages/GillespieSSA/index.html.

[46] Chao DL, Halloran ME, Obenchain VJ, Longini JrIM (2010). FluTE, a publicly available stochastic influenza epidemic simulation model. PLoS Comput Biol.

[47] Van den Broeck W, Gioannini C, Gonçalves B, Quaggiotto M, Colizza V, Vespignani A (2011). The gleamviz computational tool, a publicly available software to explore realistic epidemic spreading scenarios at the global scale. BMC Infect Dis.

[48] Falenski A, Filter M, Thöns C, Weiser AA, Wigger J-F, Davis M, Douglas JV, Edlund S, Hu K, Kaufman JH (2013). A generic open-source software framework supporting scenario simulations in bioterrorist crises. Biosecurity bioterrorism: biodefense Strateg Pract Sci.

[49] Grefenstette JJ, Brown ST, Rosenfeld R, DePasse J, Stone NTB, Cooley PC, Wheaton WD, Fyshe A, Galloway DD, Sriram A (2013). Fred (a framework for reconstructing epidemic dynamics): an open-source software system for modeling infectious diseases and control strategies using census-based populations. BMC Public Health.

