# Correction to: The Kendrick modelling platform: language abstractions and tools for epidemiology

**DOI:** 10.1186/s12859-019-2986-z

**Published:** 2019-08-27

**Authors:** Mai Anh BUI T, Nick Papoulias, Serge Stinckwich, Mikal Ziane, Benjamin Roche

**Affiliations:** 1 Software Engineering Department, School of Information and Communication Technology, Hanoi University of Science and Technology, Hanoi, Vietnam; 2 Université de La Rochelle, UMR 7266 LIENSs, CNRS, La Rochelle, France; 3 Sorbonne Université, IRD, Unité de Modélisation Mathématiques et Informatique des Systèmes Complexes, UMMISCO, F-93143 Bondy, France; 4 Université de Yaoundé I, IRD, UMMISCO, Yaoundé, Cameroon; 5 Université de Caen Normandie, Caen, France; 6 Université de Paris, Paris, France; 7 Sorbonne Université, CNRS, Laboratoire d’Informatique de Paris 6, LIP6, F-75005 Paris, France

**Correction to: BMC Bioinf (2019) 20:312**

**https://doi.org/10.1186/s12859-019-2843-0**

Following publication of the original article [1], the author noticed that the following lines were missing from the published article. The original article has been corrected.

In the ‘Results’ section under heading ‘Case study I: Measles’, the following lines are missing in the published article:

1. line ‘KendrickModel SEIR’ should be inserted before the following:

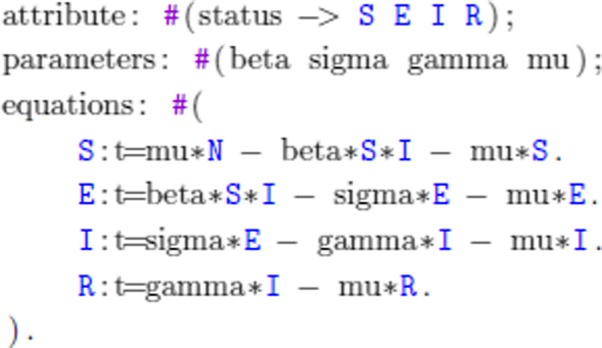



2. line ‘Simulation MeaslesRKSim rungeKutta’ should be inserted before the following:

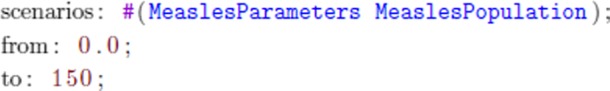

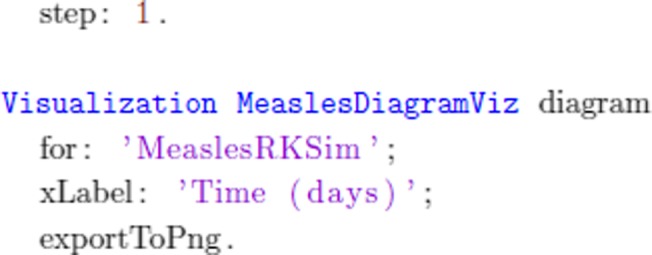





3. line ‘KendrickModel SIR’ should be inserted before the following:

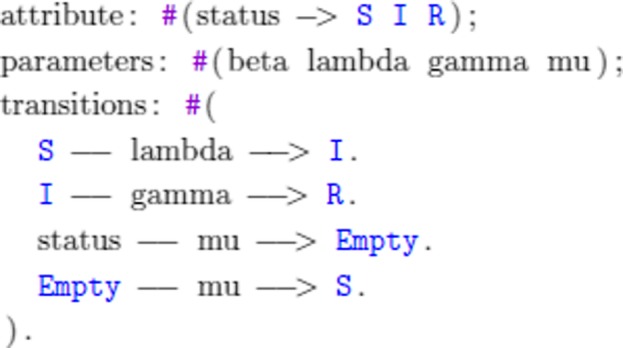



4. line ‘Experiment PopRateAnalysis’ should be inserted before the following:


