# Supplementary material for: The Kendrick modelling platform: language abstractions and tools for epidemiology
Source: BMC Bioinformatics. 2019 Jun 11;20:312. doi: 10.1186/s12859-019-2843-0 (PMC6560906; doi:10.1186/s12859-019-2843-0)
Supplement: Supplementary file 1 — A spatial SIR model specified with Kendrick. (PDF 348 kb) [file 12859_2019_2843_MOESM1_ESM.pdf]

A spatial SIR model specified with Kendrick

BUI Thi Mai Anh[1], Nick Papoulias[2], Serge Stinckwich[3,4,5], Mikal Ziane[6,7], Benjamin Roche[3]

[1] Software Engineering Department, School of Information and Communication Technology, Hanoi University of Science and Technology
[2] UMR 7266 LIENSs, CNRS, Université de La Rochelle, France
[3] Sorbonne Université, IRD, Unité de Modélisation Mathématiques et Informatique des Systèmes Complexes, UMMISCO, F-93143, Bondy, France
[4] Université de Yaoundé I, IRD, UMMISCO, Yaoundé, Cameroon
[5] Université de Caen Normandie, Caen, France
[6] Université de Paris, Paris, France
[7] Sorbonne Université, CNRS, Laboratoire de Paris 6, LIP6, F-75005 Paris, France

1 The SIR model with spatial dynamics

Here we investigate an example of a meta-population model to demonstrate a spatial concern. Assuming that the studied disease is based on the SIR model of epidemiology described by the following system of equations:

$$\begin{cases} \frac{dS}{dt} = -\beta SI \\ \frac{dI}{dt} = \beta SI - \gamma I \\ \frac{dR}{dt} = \gamma I \end{cases} \tag{1}$$

The model is simplified by ignoring demographical effects. The spatial concern groups the individuals into n countries and allows movement between neighbour countries. In our example, we consider the disease transmission between six African countries ($n = 6$). As an individual can move between neighbour countries, the population size of each country N_p is not constant.

The mobility equation for the country p is:

$$\frac{dN_p}{dt} = \sum_{q=1}^{n} \rho_{pq} N_q - \sum_{q=1}^{n} \rho_{qp} N_p \tag{2}$$

where ρ is a matrix of which each element ρ_{pq} denotes the migration rate of individuals from q to p. The first sum in the right-hand side of Equation 2 accounts for immigration to p while the second sum accounts for emigration from p.

Applying this spatial concern to an SIR model splits the compartments into p countries. Due to the spatial heterogeneities, the parameters β, γ of the SIR model are expected to be different for each country. γ then becomes a vector to describe the intra-infection between individuals in a country and β a matrix to

represent the inter-infection between countries. As an example to demonstrate a meta-population model of epidemiology, we just assume that the parameters β, γ have same value for all countries and the individuals move between countries with the same rate ρ. The inter-infection between countries is only caused by the immigration/emigration between countries.

2 Using Kendrick to specify the model

The model is specified in the Kendrick language as follows.

```
KendrickModel SIR
  attribute: #(status -> S I R);
  parameters: #(beta lambda gamma);
  transitions: #(
    S -- lambda --> I.
    I -- gamma --> R.
  );
  lambda: #(beta*I).

Map SixCountriesInAfrica
  for: #(country -> Senegal Guinea SierraLeone Liberia
          IvoryCoast Mali);
  borders: #(
              #(0 1 0 0 0 1)
              #(0 1 1 1 1 1)
              #(0 1 0 1 0 0)
              #(0 1 1 0 1 0)
              #(0 1 0 1 0 1)
              #(1 1 0 0 1 0)
          ).
KendrickModel Spatial
        maps: 'SixCountriesInAfrica';
        withTransitionRate: #(rho).

Composition SIRSpatial
        model: 'SIR';
        model: 'Spatial';
        lambda: #(beta*I_country).

Scenario SIRSpatialParams
        on: 'SIRSpatial';
        beta: 0.0002;
        gamma: 0.1;
        rho: 0.05;
        populationSize: 6000;
        S_country: #(1000 950 1000 1000 1000 1000);
```

```
37          I_country: #(0 50 0 0 0 0).
38
39  Simulation SIRSpatialSim rungeKutta
40          scenarios: #(SIRSpatialParams);
41          from: 0.0;
42          to: 100;
43          step: 0.1.
44
45  Visualization SIRSpatialViz1 map
46          for: 'SIRSpatialSim';
47          data: #(country I_country peakOfEpidemic);
48          exportToPng.
```

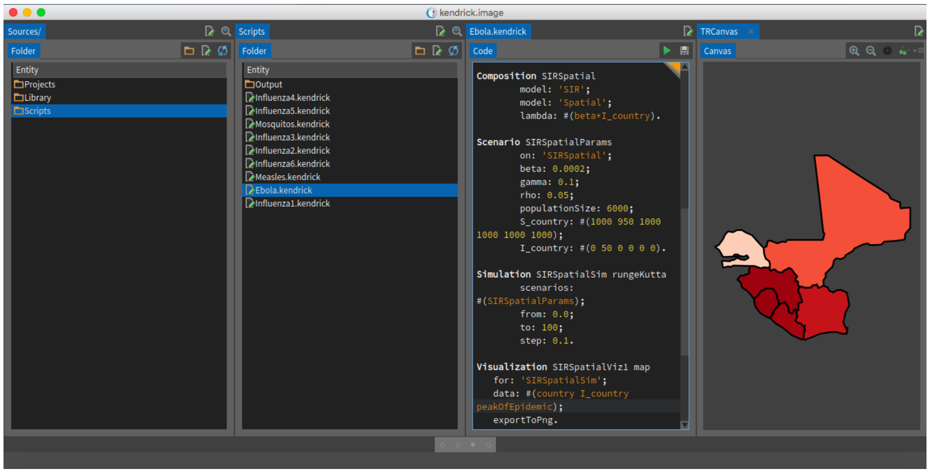

Fig. 1. The script and map view of the epidemic peak in six countries. As the disease starts from Guinea then propagates to other countries via mobilities of hosts, the colour of Guinea is the most prominent.

In this script, we define two concerns: the SIR concern (lines 1 to 8) and the spatial concern (lines 21 to 23). The model represents the six following countries in Africa: Senegal, Guinea, Sierra Leone, Liberia, Ivory Coast and Mali. Spatial maps are represented as a set of nodes (spatial elements, i.e., countries) and edges (borders between elements). Such a graph is represented using an adjacency matrix with rows and columns labeled by graph vertices $\{v_i\}$, with a 1 or 0 in position (v_i, v_j) depending on whether v_i and v_j are adjacent or not. In this example, we define our spatial concern by importing the map of six countries from its graph adjacency matrix (lines 10 to 20). Running the example using the deterministic simulation will produce the results seen in Figure 1. The colour of

each country depends on the maximum number of infections in this country (i.e. the peak of epidemic).

THe following visualisation entity will produce the infectious dynamics in each country (Figure 2):

```
Visualization SIRSpatialViz2 diagram
  for: 'SIRSpatialSim';
  data: #(I_country);
  legendTitle: 'Total of Infectious';
  legends: #('Senegal' 'Guinea' 'SierraLeone' 'Liberia'
             'IvoryCoast' 'Mali');
  xLabel: 'Time (days)';
  exportToPng.
```

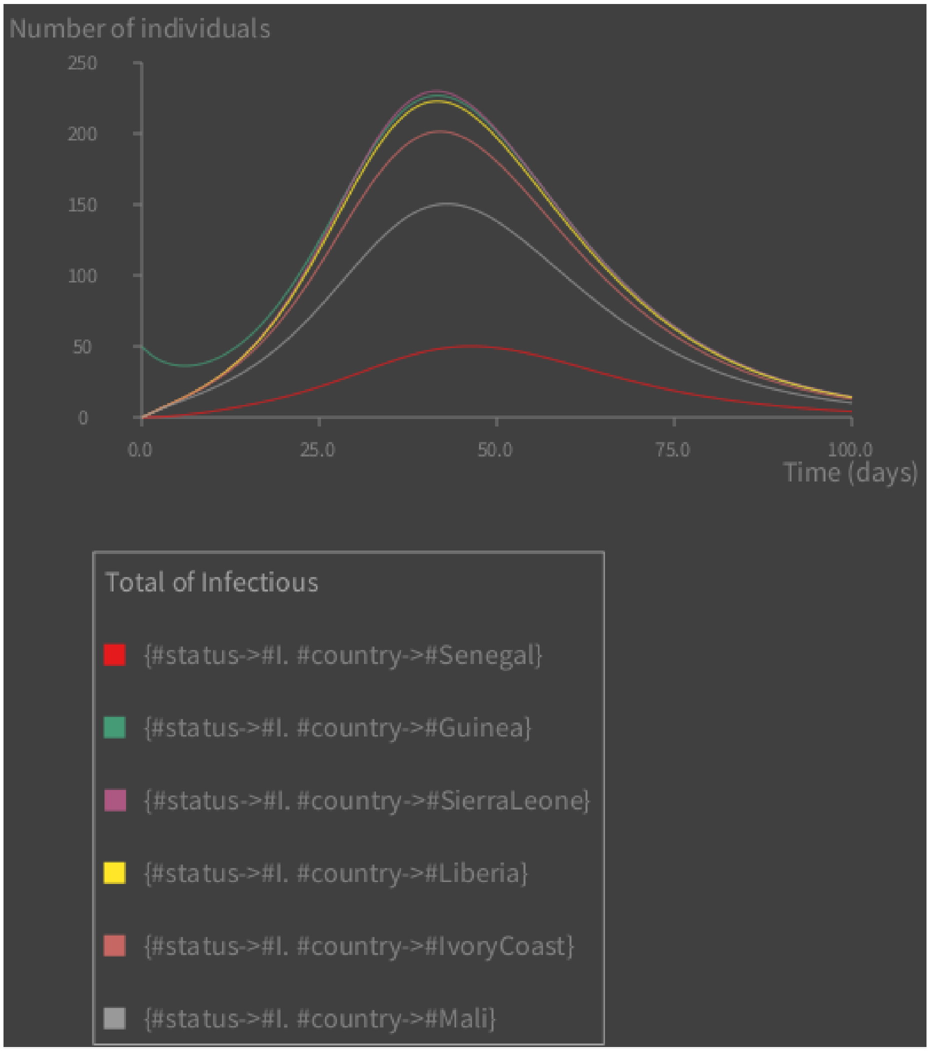

Fig. 2. The infectious dynamics in each country
